# NOD1 and NOD2: Essential Monitoring Partners in the Innate Immune System

**DOI:** 10.3390/cimb46090561

**Published:** 2024-08-28

**Authors:** Zhenjia Li, Dejing Shang

**Affiliations:** 1 School of Life Science, Liaoning Normal University, Dalian 116081, China; 2 Liaoning Provincial Key Laboratory of Biotechnology and Drug Discovery, Liaoning Normal University, Dalian 116081, China

**Keywords:** NOD1, NOD2, innate immunity, inflammatory response

## Abstract

Nucleotide-binding oligomerization domain containing 1 (NOD1) and NOD2 are pivotal cytoplasmic pattern-recognition receptors (PRRs) that exhibit remarkable evolutionary conservation. They possess the ability to discern specific peptidoglycan (PGN) motifs, thereby orchestrating innate immunity and contributing significantly to immune homeostasis maintenance. The comprehensive understanding of both the structure and function of NOD1 and NOD2 has been extensively elucidated. These receptors proficiently recognize an array of damage-associated molecular patterns (DAMPs) as well as pathogen-associated molecular patterns (PAMPs), subsequently mediating inflammatory responses and autophagy. In recent years, emerging evidence has highlighted the crucial roles played by NOD1 and NOD2 in regulating infectious diseases, metabolic disorders, cancer, and autoimmune conditions, among others. Perturbation in either their loss or excessive activation can detrimentally impact immune homeostasis. This review offers a comprehensive overview of the structural characteristics, subcellular localization, activation mechanisms, and significant roles of NOD1 and NOD2 in innate immunity and related disease.

## 1. Introduction

Nucleotide-binding oligomerization domain (NOD)-like receptors (NLRs) are crucial pattern-recognition receptors (PRRs), located within the cytoplasm to fortify innate immunity [1,2]. These versatile proteins can detect various pathogens and their associated by-products, ranging from fungi and bacteria to viruses, serving as frontline sentinels that trigger a series of signaling pathways [1,2,3]. The activation of NLRs initiates a cascade of events, encompassing the activation of innate immunity, culminating in the secretion of pro-inflammatory cytokines and chemokines that eliminate infections and damaged cells [4,5]. Both inactivation and over-activation of NLRs can compromise the balance of the host immune system and precipitate pathological conditions. NLRs are expressed ubiquitously across diverse taxa, including bacteria, fungi, algae, plants, animals, etc. [6]. Currently, 23 NLRs have been found in humans, 34 have been found in mice, and 405 have been found in zebrafish [6,7]. These evolutionarily conserved proteins have undergone positive selection and diversified among different species. This enables NLRs to recognize distinct ligands and initiate host-specific responses that subsequently trigger unique cascade reactions.

**Figure 1:**
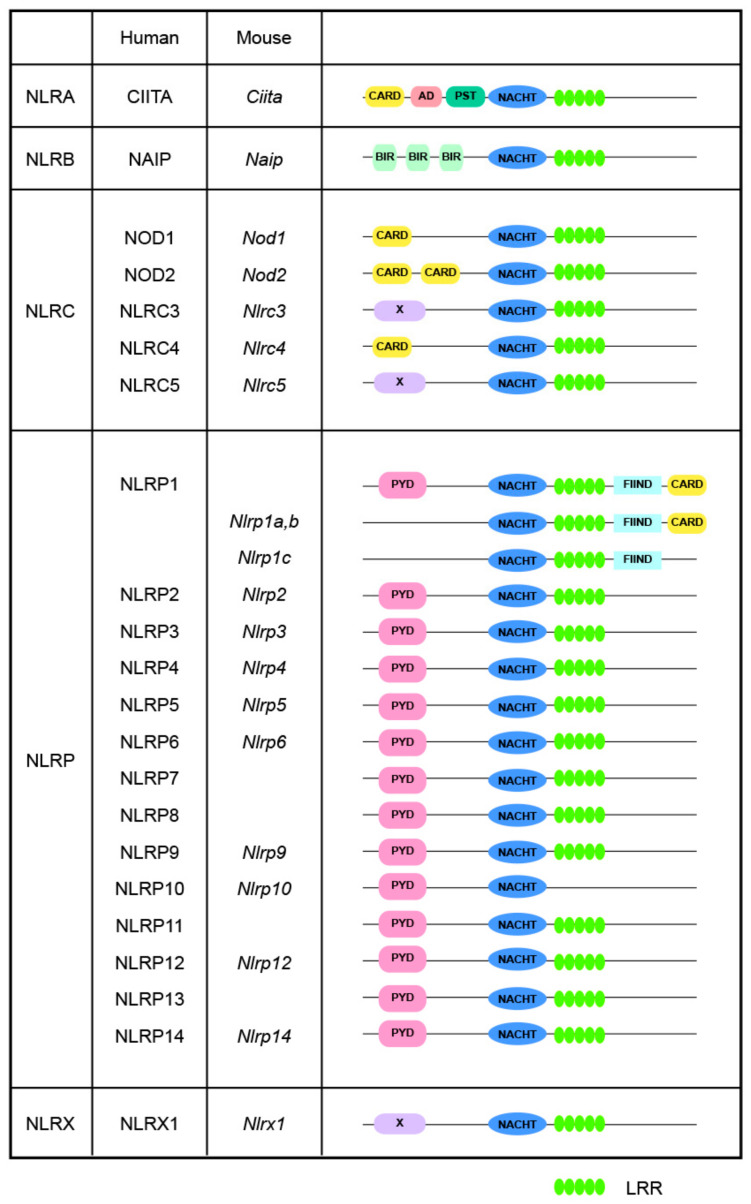
Structures of NLRs. Human and mouse NLRs are classified into five distinct subgroups based on their N-terminal domains: NLRA contains an acidic transactivating domain (AD); NLRB, also known as NAIPs, which contains a baculovirus inhibitor of apoptosis protein repeat (BIR); NLRC, which contains a caspase activation and recruitment domain (CARD); NLRP, which contains a pyrin domain (PYD); and NLRX, which possesses a unique domain with no similarity to known NLR subfamily members. NLRC1 and NLRC2 are commonly referred to as NOD1 and NOD2, respectively. Abbreviations: CIITA: MHC class II transcription activator; FIIND: autoproteolytic domain; LRR: leucine rich repeat; NAIP: NLR family apoptosis inhibitory protein; PST, proline/serine/threonine.

In mammals, most NLR proteins are composed of three core domains: (1) an N-terminal variable effector domain for downstream signaling transduction; (2) a central nucleotide-binding domain (NBD) for oligomerization, the NACHT domain; and (3) a C-terminal leucine-rich repeat (LRR) domain. The NACHT domain is composed of four sub-domains, the NBD, the helical domain 1 (HD1), the winged-helix domain (WHD), and the helical domain 2 (HD2), which are necessary for ATPase activity [6,8]. As shown in Figure 1, based on the unique N-terminal variable effector domains they harbor, NLRs are classified into four subfamilies, namely the NLRA, NLRB, NLRC, and NLRP subfamilies, corresponding to the acidic transactivation domain of CIITA, baculovirus inhibitor of apoptosis repeat domain (BIR), pyrin domain (PYD), and caspase activation and recruitment domain (CARD), respectively. Furthermore, the NLRX subfamily exhibits no significant homology with any known N-terminal domain; thus far, the sole identified member of this subfamily is NLRX1 [9]. These different N-terminal domains determine the functional repertoire exhibited by NLRs [10], enabling them to trigger diverse downstream cascades, thereby promoting the comprehensivity and adaptability of the immune system [11].

As the earliest identified and characterized mammalian NLRs, NOD1 and NOD2 serve as prominent members within the NLRC subfamily. They predominantly sense peptidoglycans (PGN), which are conserved microbial motifs abundant in bacterial cell walls [5]. NOD1 and NOD2 play a crucial role in maintaining immune homeostasis, particularly within the gastrointestinal tract, thereby significantly contributing to the overall resilience of immune defense [12]. The activation of NOD1 and NOD2 initiates a sophisticated cascade of events that ultimately modulate cellular processes essential for optimal immune function [4,13]. By delving into the intricacies of their activation, signaling pathways, and downstream influences, this review offers an overview of the regulatory roles executed by NOD1 and NOD2 related to host innate immunity.

## 2. Basic Structure

**Figure 2:**
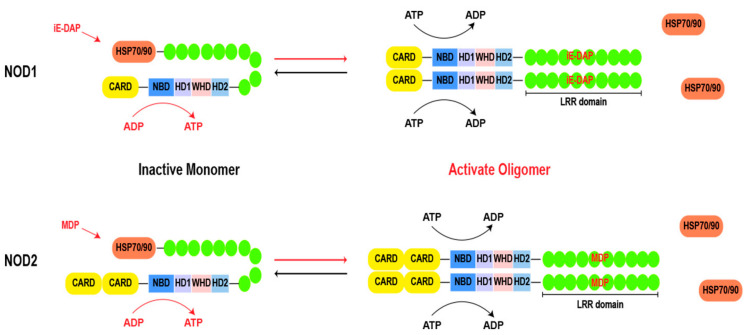
Basic structure of NOD1 and NOD2. During the resting state, the chaperone HSP70 or HSP90 stabilizes the LRR of NOD1 and NOD2, while the bent LRRs cover the NACHT domain. Upon ligand stimulation, LRR expends to recognize the ligand, leading to a transition from an autoinhibitory state to a semi-open conformation and exposing the NACHT domain. Subsequently, a nucleotide-catalyzed reaction (from ADP to ATP) occurs in the NBD domain, resulting in oligomerization of NOD molecules and recruitment of downstream effector molecules for signal transduction. Finally, ATP hydrolysis leads to inactivation and reset of the signaling platform. Abbreviations: HSP 70: heat shock protein 70; HSP 90: heat shock protein 90; CARD: caspase activation and recruitment domain; LRR: leucine rich repeat; NBD: nucleotide-binding domain; HD1: helical domain 1; HD2: helical domain 1; WHD: the winged-helix domain; MDP: muramyl dipeptide; iE-DAP: γ-D-glutamyl-meso-diaminopimelic acid dipeptide.

Both NOD1 and NOD2 share a common structural framework. As shown in Figure 2, the structure begins with the C-terminal LRR domain, which functions as a ligand sensor. Moving towards the central core, the NACHT domain acts as a pivotal region for oligomerization [14,15]. Within the NACHT domain, there is an ATP/GTPase-specific P-loop and Mg^2+^-binding site (Walker A and B motifs), collectively forming the NBD, which is indispensable for ATP binding and hydrolysis. Additionally, the NACHT domain comprises proximal HD1, distal HD2, and WHD regions [13,16]. These motifs govern conformational changes for downstream signaling. At the N-terminal, the CARD is known for its role in recruiting downstream effector proteins. This region acts as a signaling hub, triggering cascades after ligand recognition [17]. NOD1 contains a CARD, and NOD2 possesses two tandemly arranged CARDs.

In the absence of ligands, chaperones, such as heat shock protein 70 (HSP70) or heat shock protein 90 (HSP90), play a crucial role in stabilizing the monomeric structure of NOD1 and NOD2 [18,19]. The chaperones maintain the dormancy of the proteins until a pathogenic invasion prompts their separation [19,20]. Upon recognition of their respective ligands, NOD1 and NOD2 undergo a conformational change, transitioning into a semi-opened state. The exposure of the NACHT domain provides a site for ATP binding and hydrolysis, thereby driving the homo-oligomerization of two NOD molecules [21]. The formation of homo-oligomerization structures is a hallmark of NOD1 and NOD2 activation [15]. The NACHT domain is characterized as the signal transduction ATPases with numerous domains (STAND) clade of the AAA+ ATPase superfamily, the Walker A box plays an essential role in nucleotide binding, while the Walker B box coordinates catalytic ATP hydrolysis through magnesium ion coordination. Additionally, the distal extension of the Walker B box (DGhDE), consisting of two conserved acidic residues, serves as a crucial water molecule involved in ATP hydrolysis [22,23]. Adjacent to the Walker B box is the extended Walker B box (DGhDE), which comprises two conserved acidic residues and primes the pivotal water molecule involved in ATP hydrolysis [24,25]. Any mutations in these motifs cause the disruption of the finely tuned balance inherent in these processes, leading to aberrant signaling and compromised immune responses [13]. In brief, the process of NOD1 activation can be delineated into the following four steps: (i) recognition of its ligand and dissociation from its chaperone; (ii) conformational changes from an autoinhibitory state to a semi-open conformation, resulting in exposure of the NACHT domain; (iii) the nucleotides undergo a catalytic reaction (from ADP to ATP), leading to oligomerization of NOD1 and recruitment of downstream effectors for signal transduction; and (iv) ATP hydrolysis leads to inactivation and reset of the signaling platform. It is worth noting that, in contrast to NOD1, an additional hydrolysis step is required for the inactivation of NOD2, which is facilitated by the presence of the second acidic residue within the extended Walker B box [15,26]. However, the direct interconversion of ADP and ATP can modulate the activity of the NACHT domain even in the absence of ligand activation [27]. The precise mechanism of the ligand-induced conformational change in NOD1 and NOD2 requires further examination.

NOD1 and NOD2, through their LRR domains, can distinguish between different ligands, enabling the immune system to mount responses tailored to the invading pathogen. The specificity of ligand recognition underscores the remarkable adaptability of NOD1 and NOD2, enabling discrimination between subtle differences in microbial components and activating appropriate signaling cascades. The universally conserved C-terminal LRR domain exhibits variations in size between NOD1 and NOD2 [11,16]. Comparing NOD1 and NOD2 mutants with homologous amino acid substitutions at the same LRR domain sites uncovered impaired recognition of bacteria due to mutant residues (D691V in NOD1 and E778K in NOD2), mutated residues (G857R and A944V in NOD1 as well as K989E and W902G in NOD2) compromising the functionality of NOD2 without impacting NOD1 [28]. Comparative genomic studies across species demonstrate a high degree of conservation, especially in the concave surface of the LRR region, underscoring its significance in producing a stable ligand binding site [29].

Vertebrate NLRs are generally believed to have originated from teleost fish [30]. In zebrafish, NLR-A1 and NLR-A2 serve as the fish orthologs of human NOD1 and NOD2, respectively [31]. Alongside the conserved NACHT domain, NLR-A1 contains a CARD at its N-terminal and nine LRR domains at its C-terminal, while NLR-A2 possesses two CARDs at its N-terminal and eight LRR domains at its C-terminal [31]. These domains, particularly the LRR domain, share a significant degree of amino acid sequence similarity with their human counterparts [32]. However, the NACHT domains of NLR-A1 and NLR-A2 do not include the Fisna domain—a unique 70-amino-acid upstream extension of the NACHT domain that is universally present in all teleost fish NLRs [30]. This finding suggests that NOD1 and NOD2 may have undergone more conservatively in vertebrates compared to other NLR receptors [7]. NLR-A1 and NLR-A2 also play roles in zebrafish innate immunity. Depletion of NOD1 or NOD2 in zebrafish via polyphenols decreases the expression of zebrafish dual oxidase (DUOX) and ROS production, leading to impaired control of systemic infection in a *Listeria monocytogenes* infection model [33]. The tissue distribution of NOD1 and NOD2 in zebrafish mirrors that observed in mice [30]. Additionally, while NOD1 was conserved in bird and amphibian genomes, NOD2 was not detected, suggesting the possible existence of alternative receptors instead [31]. In mammals, evolutionary tracing revealed that conserved residues were broadly dispersed across both NOD1 and NOD2 sequences with denser patches observed in the CARD, NACHT, and LRR domains [34].

## 3. Activation of NOD1 and NOD2

NOD1 is particularly attuned to Gram-negative bacteria, where such muropeptides are prevalent, but it also extends its surveillance to certain Gram-positive species. NOD2 requires PGN fragments containing an intact MurNac ring structure and an attached sugar to the dipeptide moiety and has been shown to directly bind muramyl dipeptide (MDP) that is broadly expressed in both Gram-positive and Gram-negative bacteria [35].

NOD2 has a broader range of recognized ligands, expanding its scope beyond bacterial components. It can be activated not only by bacterial PGN but also by viral single-stranded RNA (ssRNA) and synthetic double-stranded RNA (polyI:C) [35]. This versatility allows for NOD2 to participate in the defense against various microbial threats [36]. The activation of NOD2 is linked to its NBD and LRR domains, underscoring the relationship between different structural elements in the detection of diverse ligands [37]. Despite the fact that the LRR domains of NOD1 and NOD2 do not directly recognize lipopolysaccharide (LPS), the mRNA level of NOD1 and NOD2 was upregulated by LPS both in vitro and in vivo [38]. LPS induces up-regulation of NOD1 expression in RAW264.7 cells by down-regulating the expression of METTL3, a methyltransferase associated with N6-adenylate methylation (m6A). Depletion of METTL3 inhibits NOD1 degradation and RIPK2 mRNA mediated by the m6A binding proteins YTHDF1 and YTHDF2. These processes enhance the activation of the NOD1 pathway and promote inflammatory responses [39].

While both NOD1 and NOD2 are cytosolic receptors, their activation is linked to diverse cellular membranes, offering a spatial dimension to their immune surveillance. These membranes include the plasma membrane, bacteria-containing phagosomes, and endosomes, operating as potential entry points for bacteria and ligands [6,40,41]. The recruitment of NOD1 and NOD2 to these membranes is precise, often requiring an intact LRR domain. This underscores the essential role of this region in orchestrating membrane association. The specificity and efficiency of the immune response are associated with the proper localization of NOD1 and NOD2. Mutations in the LRR domain resulted in mis-localization, leading to impaired pathogen recognition of NOD1 and NOD2 [11]. Furthermore, post-translational modifications modulate the activation of NOD1 and NOD2. S-palmitoylation, a lipid modification catalyzed by ZDHHC5, is essential for the subcellular localization of NOD1 and NOD2 in response to pathogens [42]. This process ensures a prompt response even at extremely low pathogen concentrations. This modification improves the membrane association of NOD1 and NOD2 and is essential for an effective immune response against PGN [43]. The connection between lipid modifications and ligand recognition underscores the complexity of the cellular machinery in adjusting immune responses to diverse microbial encounters.

In addition, small Rho family G-proteins, including Rac1, Ccd42, and RhoA, are required for the assembly of NOD1 and NOD2 to the plasma membrane [44]. These Rho family members can directly interact with NOD1/NOD2 and recruit them to endosomes. Stimulation of Rho family G-proteins can induce reorganization of the cytoskeleton, leading to subsequent internalization of pathogens or PAMPs into the host cell [45,46]. In these cases, activation of G-proteins promotes an inflammatory response. Although the significance of membrane-localized small Rho GTPases in NOD1/NOD2 activation is widely acknowledged, their relationship with S-palmitoylation modification mentioned above remains unknown [37]. NOD1 is recruited to membrane ruffles through a complex comprising Rac1, Cdc42, and Hsp90 [47,48]. The co-localization of Rac1 and NOD2 at membrane ruffles suggests their interaction, while the co-localization of Rac2 and NOD2 with RIPK2 at ruffles indicates their association [49]. Notably, knockdown of Rac1 abolishes the membrane localization of NOD2 [50].

**Figure 3:**
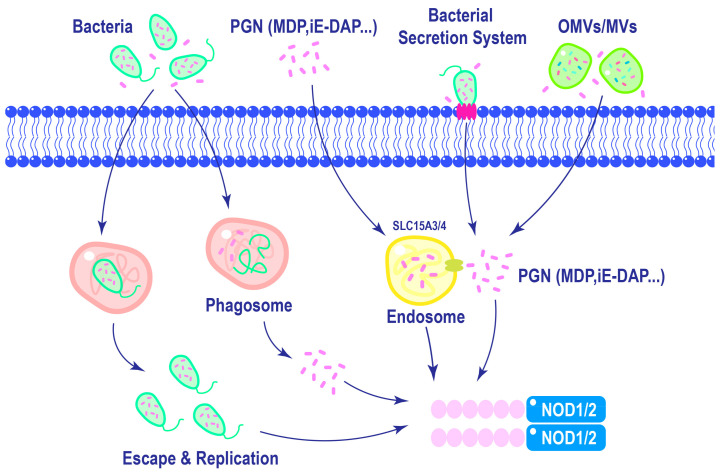
Mechanisms of NOD1 and NOD2 ligands delivery. Macrophages can phagocytose bacteria, leading to the activation of NOD1 and NOD2 in the cytoplasm upon lysosomal degradation of phagosomes. Furthermore, intracellular replication of certain pathogens can also trigger the activation of NOD1 and NOD2. In the extracellular matrix, PGNs are internalized into cells through endocytosis and subsequently delivered to NOD1 and NOD2 with the assistance of endosomal membrane transporter SLC15A3/4. Different bacterial species possess distinct secretion systems that enable direct delivery of pathogens into host cells. Additionally, Gram-negative bacteria release outer membrane vesicles, while Gram-positive bacteria like Staphylococcus aureus release membrane vesicles containing PGNs, which are internalized by cells to activate NOD1 and NOD2.

Efficient activation of NOD1 and NOD2 requires ligand delivery into the cytosol, which is a tightly regulated process. As shown in Figure 3, ligands can be internalized via approaches like phagocytosis, in which invading bacteria are engulfed by immune cells, releasing PGN into the cytosol [51]. Additionally, lysosomal digestion of polymeric PGN liberates ligands, which can engage NOD1 and NOD2 [52]. Host cells can internalize bacterial vesicles containing ligands via mechanisms like endocytosis, facilitating the delivery of microbial components to NOD1 and NOD2 [52,53]. The diversity of these mechanisms underscores the adaptability of NOD1 and NOD2 in response to diverse modes of pathogen entry. The complex relationship between ligand recognition, membrane association, and post-translational modifications emphasizes the multifaceted nature of NOD1 and NOD2 activation.

## 4. Signaling Pathways of NOD1 of NOD2

Upon recognition of their respective ligands, NOD1 and NOD2 trigger a cascade that activates inflammatory pathways and diverse functions, showcasing the complexity of their roles in host defense [13,36]. Upon ligand binding, NOD1 and NOD2 self-oligomerize through NBD and CARD. Subsequently, receptor-interacting serine/threonine-protein kinase 2 (RIPK2), also known as RIP2, is recruited to NOD1 and NOD2 [54,55,56]. RIPK2 consists of a CARD and a kinase domain. The CARD domain of RIPK2 interacts homotypically with the CARDs of NOD1 and NOD2, and the RIPK2–CARD complex undergoes accumulation and binding to produce elongated filaments, resulting in the exposure of the RIPK2 kinase domain within the outer helix structure with CARD acting as its core [55]. This conformation is stabilized by the presence of ATP. The kinase domain of RIPK2 becomes phosphorylated and ubiquitinated, but the phosphorylation process is not essential for NOD1 and NOD2 activation [57]. Ubiquitination events involving RIPK2 typically involve linear M1 ubiquitination mediated by LUBAC and K63 ubiquitination mediated by molecules like the X-linked inhibitor of apoptosis protein (XIAP). XIAP is made up of three BIR domains and three RING domains. Its BIR2 domain has a strong affinity for the kinase domain of RIPK2, enabling the ubiquitination of RIPK2 by XIAP. XIAP is a pivotal positive regulator in NOD1 and NOD2 signaling cascades [58]. Mutations in XIAP-dependent ubiquitination sites within RIP2 diminish NOD2 signaling. These events initiate a signaling cascade that produces the activation of nuclear factor-kappa B (NF-κB) and mitogen-activated protein kinase (MAPK) pathways. These pathways are essential for regulating immune responses, especially in inducing cellular inflammation [59]. The activation of NF-κB causes the transcription of pro-inflammatory cytokines, chemokines, and other immune-related genes, promoting inflammatory responses and mobilizing an array of defense mechanisms against invading pathogens.

In addition to the induction of inflammatory pathways, the activation of NOD1 and NOD2 is intricately linked to the initiation of autophagy, a cellular process with diverse implications in immune regulation. NOD1 and NOD2 directly interact with autophagy-related protein 16-1 (ATG16L1), a key component of the autophagy machinery [60,61]. This process does not involve the recruitment of RIPK2. In RIPK2-deficient cells, NOD1 and NOD2 interact with ATG16L1 along the plasma membrane [60]. However, NOD2-mediated autophagy is indispensable for both effective bacterial clearance and the production of major histocompatibility complex (MHC) class II antigen-specific CD4+ T cell responses in dendritic cells (DCs) [61]. Subsequent studies have confirmed that the NOD1/NOD2/RIPK2 pathway activates autophagy via ERK activation, resulting in the degradation of *Listeria monocytogenes* in autophagosomes. This intricate process necessitates the involvement of RIPK2 [62]. Furthermore, there is evidence supporting the involvement of RIPK2-mediated NOD2-dependent signaling in autophagy, while no role has been observed for NF-κB-mediated mechanisms [63]. The presence of ATG16L1 impedes RIPK2 polyubiquitination, restricting NOD1- and NOD2-induced NF-κB-dependent cytokine expression. Consequently, ATG16L1 is an antagonist of the classical inflammatory pathway mediated by NOD1/NOD2 [64]. The findings of these studies indicate that NOD1 and NOD2 can independently bind to ATG16L1, irrespective of RIPK2, while initiating downstream signaling in specific situations [37]. By regulating the balance between pro-inflammatory and anti-inflammatory signals, NOD1 and NOD2 contribute to the refinement of immune responses, preventing excessive inflammation and potentially leading to collateral tissue damage.

The endoplasmic reticulum (ER) contributes to protein homeostasis by regulating protein folding, processing, and transport. The accumulation of aberrant proteins can overwhelm the ER’s capacity for proper folding, producing ER stress [65]. In response to ER stress, cells activate the unfolded protein response (UPR), aiming to restore ER protein homeostasis by enhancing ER folding capacity, regulating protein translation, and triggering programmed cell death [66]. Upon activation of the unfolded protein response (UPR), inositol requiring enzyme 1α (IRE1α), an endoplasmic reticulum stress sensor, undergoes phosphorylation. This leads to the recruitment of the E3 ligase TRAF2 and kinase ASK1 to the ER membrane. The formation of IRE1α/TRAF2/ASK1 complexes activates inhibitor of nuclear factor kappa-B kinase (IKK), which phosphorylates IκB, resulting in its degradation and subsequent release of the p65 subunit (NF-kB/RelA). This released subunit translocates into the nucleus, where it stimulates the transcription of proinflammatory genes [67,68]. Sphingosine-1-phosphate (S1P) and other sphingolipid derivatives play crucial roles in regulating cellular processes, like apoptosis, proliferation, and autophagy [69]. Disturbances in cellular homeostasis result in the production of the intracellular metabolite S1P, which interacts with NOD1 and NOD2 to trigger NOD1/NOD2-dependent inflammation. S1P specifically interacts with the NBD domain of NOD1 and NOD2 without influencing its ligand binding capability. Aside from sensing microbial ligands, NOD1 and NOD2 also function as general stress sensors by monitoring cytosolic levels of the endogenous metabolite S1P [70,71].

In response to ssRNA or viral RNA treatment, NOD2 can interact with MAVS and initiate the IFN response, which was its first documented role in response to viruses [35]. Recognition of viral ssRNA by NOD2 triggers the activation of interferon regulatory factor (IRF) 3 and IRF 7, inducing type I-mediated antiviral responses [35]. This aspect of NOD2’s function highlights its contribution to antiviral immune responses. The activation of IFN-β is a critical arm of the host defense against viral infections, providing an additional layer of protection by interfering with viral replication and spread. However, the molecular recognition and signaling mechanisms underlying NOD2 receptors remain unclear due to the absence of structural similarity between bacterial MDP and viral ssRNA motifs [72]. NOD2 may be activated by directly interacting with the viral genome or proteins [35,73]. Moreover, NOD2 may collaborate with NOD1 in promoting inflammatory and antiviral responses [72]. The involvement of NOD2 in antiviral defense has been documented against respiratory syncytial virus (RSV), Zika virus, parainfluenza virus 3, vesicular stomatitis virus (VSV), influenza A virus (IAV), and Middle East respiratory syndrome (MERS-CoV) [35,74,75]. Interestingly, the antiviral effects mediated by NOD2 may be overcome by evolving viruses. Infection with the foot-and-mouth disease virus (FMDV) induced transcription of NOD2 while lowering its protein abundance. Specifically, FMDV 3Cpro cleaves various host proteins, including NOD2, limiting protein abundance. In contrast, FMDV 2B or 2C interacts with NOD2 during viral infection [76]. Although NOD1 exhibits a relatively lower responsiveness towards ssRNA compared to NOD2, it can elicit IFN signaling through MAVS in epithelial cells [77,78]. However, data regarding NOD2’s role in severe acute respiratory syndrome coronavirus 2 (SARS-CoV-2), responsible for the COVID-19 pandemic, remain limited. Preliminary evidence suggests that NOD2 may activate NF-κB and promote interferon secretion in response to SARS-CoV-2 infection, especially in severe cases [79]. However, the secretion of IL-8 induced by NOD2 agonists was lower in COVID-19 patients, indicating that NOD2 may contribute to immunosuppression after hyperinflammation in COVID-19 disease [80]. This process may be linked to its ubiquitination [81], although further research is required. The capacity of NOD1 and NOD2 to modulate both inflammatory and antiviral responses showcases their versatile role in combating a wide array of pathogens.

**Figure 4:**
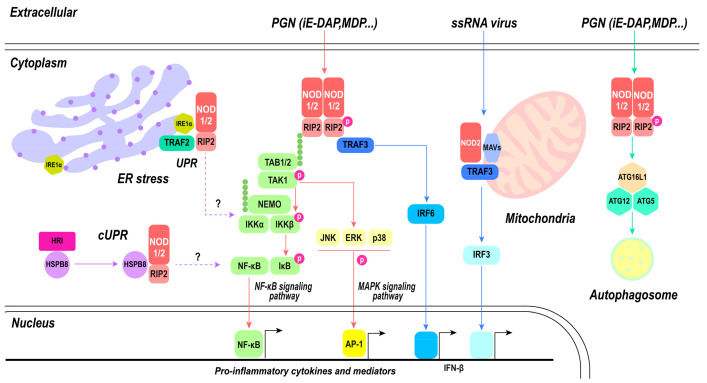
NOD1- and NOD2-mediated signaling pathway. PGN directly activates classical inflammatory pathways mediated by NOD1 and NOD2, including the NF-κB signaling pathway and MAPK signaling pathway, subsequently leading to the activation of nuclear transcription factors NF-κB and AP-1, thereby inducing the production of pro-inflammatory cytokines. Autophagy can be modulated by NOD1 and NOD2 through the activation of the ATG16L1/ATG12/ATG5 triad complex, ultimately resulting in phagosome formation. There exists an antagonistic relationship between autophagy and inflammatory pathways. Upon recognition of ssRNA, NOD2 collaborates with mitochondrial MAVs to trigger the IRF3 pathway. Both NOD1 and NOD2 play a role in maintaining cellular homeostasis. Endoplasmic reticulum stress activates inflammatory pathways mediated by NOD1 and NOD2, which are associated with IRE1α; however, the specific mechanism remains unknown. The involvement of S1P in this process is yet to be elucidated.

Overall, the signaling pathways of NOD1 and NOD2 represent a complex network integrating diverse immune responses, including inflammation, autophagy, and antiviral defense (Figure 4). The oligomerization of NOD proteins, recruitment of RIPK2, and activation of NF-κB and MAPK pathways generate the foundation of their inflammatory signaling. Simultaneously, the interaction with ATG16L1 associates NOD proteins with autophagy machinery, contributing to the regulation of inflammation and antimicrobial defense. NOD2 extends its influence on the antiviral realm by activating IRF3 and inducing IFN-β production, enhancing the host’s ability to combat viral infections. The complex and multifaceted roles of NOD1 and NOD2 underscore their importance as central players in immune system defenses against diverse pathogens. This emphasizes their potential as targets for therapeutic interventions for modulating immune responses in diverse disease contexts.

## 5. Position of NOD1and NOD2 in Innate Immunity

NOD1, ubiquitously expressed, extends its surveillance across cell types. NOD1 is highly expressed, including heart, skeletal muscle, pancreas, spleen, and ovary. Additionally, NOD1 has been detected in the placenta, lung, liver, kidney, thymus, testis, small intestine, and colon. The widespread distribution of NOD1 underscores its importance in detecting and responding to bacterial encounters in diverse tissues [82]. In epithelial cells, NOD1 activation causes the expression of chemokines and antimicrobial peptides, essential components for the recruitment of immune cells and the establishment of a hostile environment for bacteria [83]. T chemokines serve as signaling molecules, attracting immune cells, especially neutrophils, to the infection site [84]. Neutrophils, armed with potent antimicrobial mechanisms, have a central role in bacterial clearance [84]. Therefore, the activation of NOD1 in epithelial cells triggers local responses and sets in motion a cascade of events culminating in mobilizing immune defenses against bacterial invaders. In contrast, NOD2 exhibits a more restricted expression pattern, typically located in immune cells, including T cells, B cells, macrophages, DCs, and mast cells [85]. NOD2 is also highly expressed in the intestine, such as Paneth cells, stem cells, goblet cells, and enterocytes. This distribution indicates a specialization of NOD2 in cells dedicated to phagocytosis and pathogen clearance. In myeloid cells, NOD2 activation causes inflammatory cytokine production and amplification through the activation of the NF-κB and MAPK pathways [86]. Notably, NOD2 was found to be expressed in hypothalamic neurons, which has advanced our understanding of the brain–gut axis [87]. This dual effect amplifies the recruitment of immune cells to the site of infection, generating a coordinated and potent immune response against bacterial invaders [88]. The ability of NOD2 to modulate both cytokine and chemokine production stresses its versatile role in shaping the immune landscape during bacterial encounters.

Critically, coordination between NOD1 and NOD2 extends beyond their individual functions, as they form a robust defense network through interactions with Toll-like receptors (TLRs). As key components of the innate immune system, TLRs recognize PAMPs [89,90]. The cooperation between NOD1, NOD2, and TLRs is evident in experiments where ligands for TLR4 and NOD were combined, producing enhanced NF-κB activation and cytokine production relative to stimulation with individual ligands [91,92,93]. This synergy between NOD1 and NOD2 and TLRs underscores the complexity and adaptability of the immune response, enabling the host to mount a more potent defense against various pathogens [94,95,96]. The central role of NOD1 and NOD2 in host defense mechanisms is further underscored by their implications in secondary bacterial infections. In situations where TLR responses are impaired, including tolerization, the collaborative efforts of NOD receptors and TLRs are particularly vital for adequate bacterial clearance. The relationship between these receptors reflects the redundancy and synergy within the immune system, guaranteeing a robust defense against bacterial encounters.

## 6. NOD1 and NOD2 in Cancer

The development of cancer is usually affected by inflammation. Chronic inflammation facilitates tumor growth and drug resistance, while acute inflammatory responses typically induce the maturation and antigen presentation of DCs, thereby eliciting an immune response against tumors. NOD1 and NOD2 are widely expressed in immune cells and serve as crucial PRRs, demonstrating dual roles in cancer regulation [97]. NOD1 functions as a sensitizer of the TNF signaling pathway, facilitating cellular apoptosis and significantly downregulating estrogen receptor expression in breast cancer cells, thereby impeding the initiation and progression of breast cancer. This study presents the first evidence linking NOD1 to carcinogenesis; however, NOD2 does not participate in this process [98]. The upregulation of NOD1 expression is correlated with a favorable prognosis in patients with papillary thyroid cancer (PTC), as the activation of the NOD1 signaling pathway promotes PTC cell apoptosis. While in ovarian cancer, the activation of NOD1/RIPK2/NF-κB axia facilitated cell proliferation and invasion [99]. In the cervical tissues of patients with metastatic cervical squamous cell carcinoma (CSCC), NOD1 and NOD2 exhibit pronounced upregulation, thereby facilitating cancer cell proliferation and metastasis [100]. In hepatocellular carcinoma (HCC), NOD1 exerts an anti-tumor function by targeting the proto-oncogene SRC, leading to cell cycle arrest in the G1 phase and inhibition of the SRC–MAPK axis [101]. Differently, NOD2 plays a preventive role in HCC by maintaining optimal levels of cellular proliferation, immune response, and steroid metabolism [102]. Both NOD1 and NOD2 exhibit dual effects on tumor cells, which may vary depending on the tumor type and stage. In the early stages, elevated expression of NOD1 and NOD2 can activate adaptive immune responses or induce apoptosis in tumor cells. Conversely, heightened expression of NOD1 and NOD2 may exacerbate systemic inflammation by triggering an excessive release of inflammatory factors, thereby promoting tumor growth, metastasis, and invasion. NOD1 and NOD2 represent promising targets for cancer therapy, warranting a comprehensive understanding of their alterations across distinct stages and phenotypes, which holds paramount significance.

## 7. NOD1 and NOD2 in Metabolic Diseases

Metabolic diseases can be broadly characterized as a constellation of interconnected disorders that disrupt normal metabolic processes. While some metabolic diseases have a purely genetic basis, the majority are influenced by environmental factors and occasionally in conjunction with genetic predisposition. Among the prominent metabolic diseases in humans are diabetes and cardiovascular ailments. However, the term “metabolic syndrome” is increasingly employed to encompass the concurrent presence of multiple metabolic disorders, including insulin resistance, obesity, hypertension, and dyslipidemia [103]. A prominent characteristic of metabolic syndrome, diabetes, and obesity is the presence of low-grade chronic inflammation characterized by elevated levels of circulating IL-1β, IL-6, IL-18, and TNF-α [104]. The transcripts of NOD1 and NOD2 exhibited significant up-regulation and enhanced sensitivity to the recognition of their respective ligands in monocytes derived from diabetic patients. Notably, elevated expression levels of NOD1 and NOD2 were positively associated with insulin resistance [105]. Silencing of NOD2 can ameliorate myocardial apoptosis and fibrosis induced by hyperglycemia in diabetic cardiomyopathy (DCM) mice, thereby presenting a novel potential target for DCM [106]. Enhanced NOD1 expression, rather than NOD2, in the subcutaneous adipose tissue of women with gestational diabetes compared to healthy pregnant women resulted in heightened sensitivity to iE-DAP, sustained activation of the NF-ĸB signaling pathway, and release of proinflammatory cytokines [107]. The aforementioned studies suggest that NOD1 and/or NOD2 indeed promotes the progression and pathological processes of diabetes. However, it has also been reported that NOD2 may confer benefits to type 2 diabetes in mouse models by counteracting intestinal inflammation [108]. Therefore, further investigation is warranted to elucidate the precise role and underlying mechanisms of NOD2 in diabetes. NOD1 and NOD2 were also correlated with atherosclerosis (AS). The involvement of NOD2 in the development and pathological progression of AS is mediated through its exacerbation of vascular inflammation, enhancement of lipid aggregation area, and facilitation of necrosis in mice [109]. Plaque lipid deposition and inflammatory infiltration in atherosclerotic plaques exhibit an association with NOD2, while its deficiency disrupts gut cholesterol levels, microbiota composition, and oxidized low-density lipoprotein (ox-LDL) uptake by macrophages [110]. Although the roles of NOD1 and NOD2 in metabolic diseases have been elucidated to some extent through animal models, further investigations are warranted to unravel the precise underlying mechanisms. Moreover, it is imperative to comprehend the correlation between augmented or diminished expression of NOD1 and/or NOD2 and disease.

## 8. NOD1 and NOD2 as Potential Therapeutic Targets

Given their roles in immune activation, NOD1 and NOD2 have been identified as potential therapeutic targets for a range of diseases, especially those involving inappropriate or excessive inflammation. Upon recognizing their ligands, NOD1 and NOD2 activate downstream signaling pathways, particularly the NF-κB and MAPK pathways, which lead to the production of pro-inflammatory cytokines and other immune responses. This process is crucial for the body’s defense against bacterial infections. Therefore, modulating the activity of NOD1 and NOD2 may potentiate immune response against infections or mitigate detrimental hyperinflammation in certain infectious diseases. Genetic deficiency of NOD2 plays a protective role during *Aspergillus* infection. The absence of NOD2 in monocytes and macrophages enhances phagocytosis, leading to increased fungal killing; conversely, activation of NOD2 reduces the antifungal potential of these cells [111]. In septic shock, the excessive activation of NOD1 and NOD2 leads to diverse organ injury/dysfunction in animal models [112].

Mutations and single nucleotide polymorphisms (SNPs) in NOD2 confer genetic susceptibility to a range of autoimmune and autoinflammatory disorders, including Crohn’s disease, Blau syndrome, and Yao syndrome. NOD2 SNPs represent the most potent known genetic risk factor for Crohn’s disease development; however, the precise mechanism by which NOD2 variants contribute to disease pathogenesis remains incompletely understood. Mutations in NOD2 associated with Crohn’s disease are predominantly confined to the LRR domain and have been demonstrated to abolish MDP detection and NF-κB activation [113]. Furthermore, deficiency of NOD2 in Paneth cells impedes antimicrobial peptide secretion [114], thereby resulting in a functionally impaired phenotype characteristic of Crohn’s disease-associated mutations in NOD2. In contrast, Blau syndrome is characterized by gain-of-function mutations primarily concentrated within the NBD region of NOD2, leading to excessive activation of NOD2 and subsequent NF-κB activation independent of MDP [25,115]. In addition, NOD2 harbors two crucial S-palmitoylation sites, and several NOD2 mutant proteins associated with Crohn’s disease exhibit reduced palmitoylation levels. However, the Blau syndrome mutation NOD2^C495Y^ series demonstrates an elevated level of S-palmitoylation. Overall, both hypo- and hyperpalmitoylation of NOD2 contribute to aberrant signaling [37].

Overall, small molecule inhibitors that block NOD1 or NOD2 signaling could be used to treat diseases characterized by excessive inflammation; on the other hand, NOD1 or NOD2 agonists could be employed to enhance the immune response against certain infections or even in cancer therapy to boost anti-tumor immunity. The identification of drugs that selectively target NOD1 or NOD2 while minimizing interference with other immune pathways is imperative to mitigate potential side-effects. Additionally, achieving a delicate equilibrium in modulating the activity of NOD1 or NOD2 is essential. It is also crucial to consider the impact of genetic variations in NOD1 and NOD2 among individuals on both the efficacy and safety of potential therapies.

## 9. Discussion

NOD1 and NOD2 are sentinel sensors, foundational to innate immunity, that recognize peptidoglycan and respond to cellular stress. These receptors play a pivotal role in orchestrating an array of signaling pathways essential to both immune responses and tissue repair mechanisms, contributing to the maintenance of health and the enhancement of resistance against infectious and inflammatory diseases. Efficient activation of NOD1 and NOD2 requires the delivery of ligands to the cytosol, a tightly regulated process. Ligands can be internalized via phagocytosis, leading to the release of PGN into the cytosol. Additionally, lysosomal digestion of polymeric PGN contributes to the liberation of ligands, potentially activating NOD1 and NOD2. Host cells can also internalize bacterial vesicles containing ligands via endocytosis, enabling the delivery of microbial components to NOD1 and NOD2. The diversity of these mechanisms underscores the adaptability of NOD1 and NOD2 in responding to diverse modes of pathogen entry. The complex relationships between ligand recognition, membrane association, and post-translational modifications highlight the multifaceted nature of NOD1 and NOD2 activation. Ligand-induced conformational changes, enabled by homo-oligomerization and ATP hydrolysis, trigger downstream signaling pathways. The involvement of chaperones, specificity in ligand recognition through LRR domains, and subcellular localization emphasize the precision and versatility of NOD1 and NOD2 in detecting and responding to microbial threats. Upon recognizing their respective ligands, NOD1 and NOD2 trigger a cascade of events activating inflammatory pathways with diverse functions, showcasing their complex roles in host defense. The recruitment of RIPK2 to activated NOD1 and NOD2 is a crucial step in the signaling pathway. RIPK2 undergoes phosphorylation and ubiquitination, initiating a signaling cascade culminating in the activation of the NF-κB and MAPK pathways. These pathways regulate immune responses via cellular inflammation. In addition to inducing inflammatory pathways, NOD1 and NOD2 activation is associated with autophagy initiation. Direct interactions with ATG16L1, a crucial component of the autophagy machinery, highlight the multifunctional roles of NOD1 and NOD2. Furthermore, the endoplasmic reticulum (ER) is vital in NOD1 and NOD2 signaling. The interactions with IRE1α and S1P highlight the integration of NOD1 and NOD2 into various cellular processes beyond immediate immune responses. In response to viral RNA treatment, NOD2 can interact with MAVS and IFN responses. This extends the role of NOD2 into antiviral defense, producing type I-mediated antiviral responses. The cooperation between NOD1 and NOD2 in promoting inflammatory and antiviral responses emphasizes their versatile role in combating various pathogens.

Moreover, gaining insights into the context-specific functions of NOD1 and NOD2 is essential for tailoring therapeutic approaches to various disease scenarios. The diversity of ligands and cellular contexts in which these receptors operate requires a nuanced comprehension of their roles in specific diseases. The therapeutic implications of targeting NOD1 and NOD2 are promising. Their central roles in modulating immune responses make them possible targets for therapeutic interventions to regulate inflammation in diverse disease contexts. However, the complex nature of their signaling pathways and the need for a nuanced understanding of their roles in specific diseases require further examination.
